# The Army Medical Bill

**Published:** 1908-04

**Authors:** 


### The Army Medical Bill

AT last, after many weary months of waiting, the army medical department has come into its own—nearly. The house on March 16, passed the medical reorganisation bill which was sent to it from committee in amended form and there is little probability that it will be again interfered with. All that remains now is the conference report and the signature of the President, and the bill becomes of force.

In its amended form the bill provides for an army medical corps comprised of a Brigadier General, 14 colonels, 20 lieutenant colonels, 100 majors and 300 captains and first lieutenants. There is provision made for a medical reserve corps to be composed of first lieutenants.

The passage of this bill by the house, while it does not by any manner of means provide for all that is necessary for an adequate corps so far as its numerical strength is concerned, does provide a better basis upon which to build a corps that shall be sufficiently strong in numbers to properly care for the sick and wounded of an army. The concession of the congress in giving the medical corps even a portion of what was asked for, and which is so apparently needed for the proper performance of medical service, gives rise to the hope that in the no distant future a perfect organisation may be effected, on the lines laid down by the Surgeon General in his original estimate which was by no means exaggerated. However, the medical department has gained something and for that something let us be thankful; for it gives promise that with continued agitation by the profession at large we may yet bring the political mind to take a common sense view of the situation as it exists and see the lack of wisdom in freely spending millions in building up a great fighting machine and in methods of offense and defense, and grudgingly doling out pennies for the protection of the health and physical well being of that splendid machine.

One of the most pleasing features of the bill, and one that is indicative of the awakening of the political mind, is that section which provides for the establishment of a medical reserve corps, to be made up of commissioned first lieutenant surgeons. It is specifically provided that the members of this reserve corps cannot rise higher in rank; that they shall be paid only when on actual duty; that they shall not be entitled to retirement pay for length of service, nor to increased pay except for actual time served. They do however, take rank as actual commissioned officers, and as such are entitled to the privileges of officers as well as to pensions for disabilities actually incurred while on service and in line of duty.

This reserve corps forever does away with the disgraceful and humiliating system of giving contracts to surgeons; a system which has been as objectionable to the surgeon general of the army as it has been to the members of the corps and to those who accepted contracts.

Shortly after the Spanish war, the JOURNAL called attention to the system then in vogue, that of employing physicians under contract and ranking them as acting assistant surgeons with the relative rank of first lieutenants. They were not commissioned officers and had none of the rights of an officer although they did all the service usually performed by a commissioned medical officer except serving on courts martial and swearing in recruits. They were executive officers of hospitals; they were operators; they were investigators; and some of the most brilliant work during the war with Spain and in the Philippine insurrection was done by acting assistant surgeons under the direction and guidance of regular army surgeons. At that time the suggestion was made in the JOURNAL that the position of the acting assistant surgeon could be made less humiliating by the organisation of such a corps as has now been provided for, and its members given commissions. Instead, the change made was in the opposite direction and the acting surgeon was merely designated officially as a "contract surgeon."

Happily, all this will be changed under the new order of things and it will not be long, it is hoped, before there will be, in addition to the increased medical corps provided for in the bill, a medical reserve corps composed, in the first instance, of men who have had army experience; who will be available for any duty, at any time and in any place, thus placing at the disposal of the surgeon general in the event of necessity a thoroughly capable, well-trained body of medical men who will go into service under commission and with actual rank commensurate with the dignity of their profession; men whom it will not be necessary, as it was in the Spanish war days, to lead by the hand through the mazes of military medicine and executive work.

An added advantage of the establishment of the reserve corps is that it may be made possible for the surgeon general to have at his command an almost unlimited number of experienced specialists in the various branches of medicine and surgery—an invaluable adjunct in the event of war.

Now that an opening has been made into the hitherto closed political conscience—and it is not a wholly selfish one, by any means—the work for betterment should not cease. Consistent, earnest effort toward the improvement of the medical corps in numerical strength will assuredly result in bringing the department up to a standard where it will be fully prepared to meet any emergency which may arise.

And the surgeon general should have the rank of major general.

